# Low dietary magnesium and fiber intakes among women with metabolic syndrome in Kuwait

**DOI:** 10.3389/fnut.2024.1451220

**Published:** 2024-10-16

**Authors:** Dalal U. Z. Alkazemi, Tasleem A. Zafar, Nourah Y. Alsouri, Abeer A. Aljahdali, Stan Kubow

**Affiliations:** ^1^Department of Food Science and Nutrition, College of Life Sciences, Kuwait University, Al-Shadadiya, Kuwait; ^2^Department of Clinical Nutrition, Faculty of Applied Medical Sciences, King Abdulaziz University, Jeddah, Saudi Arabia; ^3^Department of Nutritional Sciences, School of Public Health, University of Michigan, Ann Arbor, MI, United States; ^4^School of Human Nutrition, McGill University, Montréal, QC, Canada

**Keywords:** metabolic syndrome, dietary magnesium, dietary fiber, Kuwait, women, nutritional epidemiology

## Abstract

**Introduction:**

Metabolic syndrome (MetS) is a growing health concern among Kuwaiti women. Dietary magnesium and fiber have been implicated in reducing the risk of MetS; however, their specific effects on this population remain underexplored. This study aimed to investigate the association of dietary magnesium and fiber intake with the prevalence of MetS and its components among women in Kuwait.

**Methods:**

This study included 170 women aged 18–65 (years) recruited from AL-Adan Hospital, Mubarak Hospital, and Riqqa Polyclinic. Data were collected using a modified Semi-Quantitative Food Frequency Questionnaire (SFFQ) to assess dietary intake, and biochemical measurements were performed to evaluate serum magnesium and other metabolic markers. MetS was diagnosed according to International Diabetes Federation (IDF) and Adult Treatment Panel III (ATP III) criteria. Statistical analyses included Mann–Whitney *U*-tests, chi-square tests, Spearman correlations, logistic and linear regression models, and Cohen’s kappa statistics.

**Results:**

The prevalence of MetS was 24 and 18% based on the IDF and ATP III criteria, respectively. Women with MetS had significantly lower dietary magnesium and fiber intakes than those in women without MetS (*p* < 0.001). A strong positive correlation was found among dietary magnesium intake, fiber intake, and serum magnesium levels (*r* = 0.957, *p* < 0.001 for magnesium; *r* = 0.917, *p* < 0.001 for fiber). Increased dietary magnesium and fiber intakes were linked to reduced odds of developing MetS and its components, except for blood pressure measurements. Cohen’s kappa demonstrated a strong agreement (*K* = 0.70, *p* < 0.001) between dietary and serum magnesium inadequacy.

**Conclusion:**

Increased dietary intakes of magnesium and fiber are associated with reduced odds of developing MetS among Kuwaiti women. These findings support the promotion of magnesium- and fiber-rich diets as preventive strategies against MetS.

## 1 Introduction

Metabolic syndrome (MetS) is a global health concern characterized by interconnected metabolic abnormalities, including abdominal obesity, dyslipidemia, hypertension, and impaired glucose metabolism. This syndrome not only poses a significant threat to cardiovascular health but also increases the risk of type 2 diabetes and other chronic diseases (1, 2). In the Middle East, particularly in Kuwait, the rising prevalence of MetS is a growing public health concern.

Alarmingly, statistics in Kuwait reveal that approximately 37.7% of women aged 20 years or older have MetS, as assessed using the United States National Cholesterol Education Program Adult Treatment Panel III (ATP III) criteria (3). This rate increases to 40.1% when assessed with the International Diabetes Federation (IDF) criteria (4). A recent study of apparently healthy Kuwaiti adults found that the prevalence of MetS was 22.1% according to the IDF criteria and 15.2% according to the ATP III criteria (5). The prevalence was higher among women, with 23.3% meeting the IDF criteria compared to 15.8% of men, indicating a persistent public health concern. Additionally, hypomagnesemia was significantly associated with MetS and its components in Kuwaiti adults (5).

Rapid economic growth in Kuwaiti society has led to profound lifestyle changes, including significant alterations in dietary habits. Traditional diets, once based on whole foods, have shifted toward a high consumption of refined carbohydrates and heavily processed foods. The current Kuwaiti diet is characterized by high caloric, cholesterol, and sodium content, with low levels of fiber, omega-6, omega-3, vitamins, and essential minerals such as calcium, magnesium, and zinc (6). Refined grains, which dominate the diet, lack crucial nutrients like magnesium, fiber, and vitamin E, all of which are essential for mitigating the risk of diabetes, heart disease, and MetS (6). High adherence to a fast-food dietary pattern among Kuwaiti adults has been associated with elevated blood pressure (BP), body mass index (BMI), and waist circumference, while a refined grains/poultry dietary pattern has been linked to increased plasma glucose concentrations (7). Given these dietary trends, it is anticipated that MetS prevalence will continue to rise in Kuwait, making it imperative to explore the dietary factors contributing to this health crisis (8).

Recent studies have demonstrated significant associations between healthy lifestyle factors and the risk of MetS. In Iranian adults, higher adherence to a healthy lifestyle score (HLS), which includes non-smoking, maintaining normal body weight, engaging in physical activity, and following a healthy diet, has been linked to a reduced risk of MetS and its components (9, 10). Conversely, higher dietary and lifestyle inflammation scores are correlated with an increased MetS risk (11). Similar findings have been observed in other populations, such as in a Spanish cohort, where adherence to a healthy lifestyle score was associated with a lower risk of developing MetS (12). Studies exploring dietary patterns have further linked specific nutrient patterns with MetS risk. For instance, a mineral-based diet has been associated with healthier metabolic outcomes, while plant-sourced nutrient patterns are linked to a lower risk of MetS (13, 14).

Conversely, animal-sourced nutrient patterns are associated with an increased risk of MetS. Western dietary patterns, characterized by high fat and low fiber intake, have consistently been associated with higher MetS risk (15, 16). Research on plant-based diets also shows varying effects on MetS, with protective effects particularly noted in women and adolescents (17, 18). These studies align with our research focus, emphasizing the critical role of diet in managing MetS, particularly in regions with high MetS prevalence.

Magnesium, found in whole grains, spinach, nuts, legumes, and potatoes, is protective against insulin resistance, hypertension, and dyslipidemia (19, 20). Similarly, dietary fiber intake is associated with lower fasting blood glucose (FBG) and glycated hemoglobin A1c levels in individuals with type 2 diabetes (21). Magnesium deficiency has been linked to various metabolic disorders, including insulin resistance, type 2 diabetes, and hypertension (2, 22, 23). Furthermore, magnesium and dietary fibers often coexist in the same foods. Several studies have highlighted the inverse relationship between fiber intake and MetS prevalence. Increased dietary fiber intake has been shown to reduce the risk of obesity, type 2 diabetes, and cardiovascular diseases (24). Magnesium is an essential cofactor in multiple enzymatic reactions and acts as a direct antagonist of intracellular calcium, influencing insulin action and carbohydrate metabolism. Insulin, the principal regulator of intracellular magnesium, further underscores the importance of magnesium in metabolic health (25).

Additionally, magnesium influences insulin sensitivity, blood pressure regulation, and lipid metabolism, while fiber is well-recognized for its positive effects on weight management, glycemic control, and lipid profiles (26). An inverse association between dietary magnesium and C-reactive protein, a marker of systemic inflammation and a predictor of MetS, has also been reported, suggesting a role for dietary magnesium in mitigating MetS risks (22). Observational studies worldwide have consistently indicated that higher dietary magnesium and fiber intakes are associated with a reduced risk of MetS (27).

In this context, serum magnesium concentrations can offer insights into dietary magnesium intake. Although serum magnesium may not directly reflect dietary intake due to the body’s regulatory mechanisms, significant deviations in serum magnesium concentrations can indicate dietary imbalances (28). Serum magnesium is the predominant test healthcare providers use to assess magnesium status because it is a valid and reliable biomarker for targeted screening at a relatively low cost (29). Studies examining dietary, serum, and/or urinary magnesium data are lacking. Such studies could inform the use of biomarkers to assess magnesium intake and its potential to improve inadequate magnesium status (29). By integrating serum magnesium evaluations with conventional dietary assessments, researchers and healthcare professionals can better determine how dietary habits affect an individual’s magnesium status. This approach can also aid in assessing the efficacy of magnesium-rich foods and supplementation interventions to improve magnesium status and reduce the risk of adverse health outcomes (30). This comprehensive approach is crucial for tailoring public health strategies that encourage the consumption of magnesium- and fiber-rich foods to counteract the high prevalence of MetS in Kuwait.

Kuwait’s distinctive cultural and dietary characteristics make it a vital setting to examine the impact of dietary magnesium and fiber intakes on MetS in women. Kuwait ranks among the world’s top 10 countries with the highest obesity rates, with a particularly high prevalence of obesity among women (31, 32). Understanding the impact of dietary magnesium and fiber intakes on MetS can be pivotal for devising effective public health interventions. This study aims to examine the associations among serum magnesium concentrations, dietary magnesium intake, fiber intake, and the prevalence of MetS, thereby making a significant contribution to the existing literature (33). Furthermore, the primary dietary contributors to magnesium and fiber intakes among Kuwaiti women were identified. By examining these relationships, this study seeks to enhance our understanding of the dietary factors affecting MetS, potentially informing future public health strategies explicitly tailored for Kuwaiti women.

## 2 Materials and methods

### 2.1 Participants and recruitment

This study was conducted at the medical laboratories and outpatient clinics of AL-Adan, Mubarak Al-Kabeer Hospital, and Riqqa Polyclinic in Kuwait. Recruitment primarily focused on individuals from the Al-Adan and Mubarak Al-Kabeer hospitals, representing the Al-Ahmadi and Hawalli Governorates, respectively. Fewer participants were recruited from the Riqqa clinic, representing the Al-Ahmadi Governorate. Participation was voluntary, and participants could withdraw from the study at any time without providing reasons or impacting their medical care. Written informed consent was obtained from all participants before enrolment in the study. Ethical approval was obtained from the Kuwait University Research Sector and Ministry of Health (MOH) (protocol code [2017/546] and date of approval: April 9, 2017). All the procedures were performed per the Declaration of Helsinki.

### 2.2 Study population

From our previous study sample (5), we exclusively focused on women, totaling 193 participants. For this current study, we refined our inclusion criteria to exclude individuals who did not complete the assessment of their dietary intake, resulting in a final cohort of 170 women aged 18–65 years. These participants were recruited from regular checkups or referrals by their general physicians for medical investigations. The study’s inclusion criteria required participants to be healthy women without prior chronic disease diagnoses or prescription medications. Exclusion criteria encompassed age limits (<18 or >65 years), pre-existing diabetes or renal failure, pregnancy or nursing status, current use of hypertension and dyslipidemia medications, consumption of fiber and magnesium supplements, and inability to fast appropriately overnight (10–12 h). Regarding special diets or under and over-nutrition, the study did not include individuals who were on special diets due to chronic diseases, weight management, or other specific health conditions. This approach helped us ensure that the dietary intake data collected reflected the participants’ usual habits without the influence of specific medical or dietary interventions.

### 2.3 Survey questionnaire

The survey questionnaire gathered comprehensive sociodemographic information, covering participants’ sex, age, nationality, education level, total income, occupation, and histories of chronic disease and family illness. It also included details on physical activity, smoking habits, and the use of medications or supplements. The second part of the questionnaire included a Semi-Quantitative Food Frequency Questionnaire (SFFQ) administered by a trained dietitian to assess dietary magnesium and fiber intakes. We modified the validated SFFQ developed by Dehghan et al. (34) to capture Kuwaiti food consumption. The portion sizes were standardized for clarity. Green leafy vegetables, whole grains, and legumes were measured in cups, while nuts were measured at 1 oz (28 g) per serving. Three-dimensional food models and household measurements were used, including standard measuring cups, glasses, spoons, and photographs. The SFFQ covered 119 foods (Supplementary Figure S1) consumed over the past 12 months. Frequency of intake was measured on a 9-point scale, from “never” to “6 times per day or more.” Dietary magnesium and fiber intakes were calculated by multiplying the frequency of food unit consumption by nutrient content, referring to the United States Department of Agriculture National Nutrient Database and Nutritional Profile of Kuwaiti Composite Dishes (35). The SFFQ categorizes foods into seven groups: milk and dairy products, bread and cereals, meat and mixed dishes, fruits, vegetables, nuts, and beans. The “mixed dishes” were defined as foods that combine various food groups, such as stews, casseroles, or salads, which typically include a mix of vegetables, grains, meats, and legumes. For each food group, dietary magnesium was expressed as total mg/day, mg/1,000 kcal, and as a percentage of the total mg/day. Dietary magnesium adequacy was evaluated according to: (1) the estimated average requirement (EAR) for magnesium intake, 255 mg for females aged 19–30 years and 265 mg for those aged 31 years and above; (2) recommended dietary allowances (RDAs) for magnesium intake, 310 mg for females aged 19–30 years and 320 mg for those aged 31 years and above; and (3) body weight adjusted EAR (36) calculated based on the population-based average weight for Kuwaiti adult women (73 kg) (31), resulting in 305.14 mg and 316.82 mg, respectively. The adequacy of dietary fiber was evaluated according to the adequate intake (AI): 25 g for females aged 19–50 years and 21 g for those aged 51 years and above.

### 2.4 Anthropometric, blood pressure, and biochemical measurements

Weight was measured using digital scales with the participants wearing minimal clothing and without shoes, and a calibrated stadiometer was used to measure height with the participants standing and their shoulders in a normal position. BMI was calculated as weight in kilograms divided by height in square meters. Waist circumference was measured with light clothing, using an unstretched measuring tape, to the nearest 0.1 cm at a level midway between the inferior margin of the ribs and the superior border of the iliac crest. Blood pressure was measured three times using a standard gauge mercury sphygmomanometer on the right arm while the participants were seated and after complete rest. The mean of the three readings was used for each blood pressure measurement. The participants fasted for 10–12 h before blood samples were collected. Trained phlebotomists collected venous blood samples from the left arm through venipuncture. The participants were seated for 5 min before blood collection. Blood samples were collected in yellow-top 3.5-mL plain tubes and processed using a Rotofix 32 A or Thermo centrifuge. The processed specimens were analyzed using an automated Roche Cobas 8,000 c702 for FBG, triglyceride (TG), high-density lipoprotein cholesterol (HDL-C), low-density lipoprotein cholesterol (LDL-C), total cholesterol (TC), and serum magnesium (Mg). The manufacturer supplied all reagents. This study used the standard reference ranges recommended by the Kuwait MOH. The MOH population-specific reference ranges defined hypomagnesemia as a serum Mg level of <0.74 mmol/L (5), consistent with the cutoff value used to define hypomagnesemia in other contexts (37). We also applied the newly proposed lower reference cut-off value of <0.85 mmol/L suggested by Rosanoff et al. (38) to identify individuals at risk of chronic latent magnesium deficit. This condition represents a subclinical state characterized by a small chronic negative magnesium balance, often due to decreased dietary intake, malabsorption, or increased renal loss, which may predispose individuals to certain diseases (38).

### 2.5 MetS diagnostic criteria

The current study identified MetS based on the IDF (39) and ATP III criteria (40). The IDF criteria included high waist circumference (≥80 cm) and the presence of two or more of the following: high TG level (≥150 mg/dL or 1.7 mmol/L), reduced HDL-C level (<50 mg/dL or 1.29 mmol/L), high blood pressure [systolic blood pressure (SBP) ≥130 mmHg or diastolic blood pressure (DBP) ≥85 mmHg], and high fasting plasma glucose (FPG) level (≥100 mg/dL or 5.6 mmol/L) (39). For the ATP III criteria, we applied the modified version, diagnosing MetS when the participants met three or more of the following criteria: waist circumference (>88 cm), high TG level (≥150 mg/dL or 1.69 mmol/L), reduced HDL-C level (<50 mg/dL or 1.29 mmol/L) high blood pressure (SBP ≥ 130 mmHg or DBP ≥ 85 mmHg or using hypertension medication), and high FPG level (≥100 mg/dL or 5.6 mmol/L or using diabetes medication) (40).

### 2.6 Statistical methods

In the current study, the self-reported dietary intakes of magnesium and fiber served as the exposures, while the odds of MetS and its components were the outcomes of interest. Descriptive statistics for the study population are presented as the mean and standard deviation for continuous variables and frequency and percentages for categorical variables. Given the violation of the normality assumption for all variables, continuous data are expressed as medians and interquartile ranges (IQRs). Using the IDF diagnostic criteria, the Mann–Whitney *U*-test was used to compare the participants’ characteristics based on MetS status. The chi-square test was used to compare differences in categorical variables between the two groups. Partial Spearman’s correlation was used to examine the association between dietary and serum magnesium levels after adjusting for age, total caloric intake, BMI, and regular menses. We also conducted multivariate linear regression to assess the association between serum magnesium level as the dependent variable and dietary magnesium or fiber as the independent variable in separate models owing to the collinearity in dietary intake (41). Furthermore, using Cohen’s kappa statistic, we assessed the agreement between classifying magnesium deficiency using serum magnesium level (i.e., at 0.74 mmol/L or lower and the lower reference cut-off value of <0.85 mmol/L) and low dietary magnesium intake based on the age-specific EAR and RDA. The Kappa results were interpreted as follows: values ≤0 indicating no agreement, 0.01–0.20 as none to slight, 0.21–0.40 as fair, 0.41–0.60 as moderate, 0.61–0.80 as substantial, and 0.81–1.00 as almost perfect agreement (42). The magnitude of the relationship between cardiometabolic risk factors, serum magnesium, and dietary magnesium and fiber was examined using Spearman’s correlation. Logistic regression analyses were conducted to investigate the association between the two exposures (dietary magnesium and dietary fiber) and the odds of developing MetS and its components. Each type of exposure was assessed individually. We evaluated the differences in sociodemographic and lifestyle characteristics between individuals with and without MetS to identify covariates that needed adjustment in the logistic regression models. We only included covariates with a *p*-value of <0.05. For each exposure, Model 1 included the exposure of interest only (i.e., crude/unadjusted model). Model 2 additionally included age. In model 3, we also tested total caloric intake and regular menses in addition to model 2, and neither had any effects. Finally, Model 5 additionally included BMI. Odds ratios (ORs) and 95% confidence intervals (CIs) were presented for the logistic regression models. Sensitivity analyses were conducted to assess for outliers, but the conclusions remained consistent and statistically significant, with only minor attenuation of point estimates. Therefore, the original analyses were reported. Statistical analyses were conducted using Statistical Analysis System software (version 9.4; SAS Institute Inc., Cary, NC, United States), and significance was set at *p* < 0.05.

## 3 Results

### 3.1 Characteristics of the study participants

The sociodemographic and lifestyle characteristics of the participants are presented in Table 1. Recruitment locations included AL-Adan Hospital (56%), Mubarak Hospital (32.35%), and Rigga Polyclinic (11.76%). None of the participants reported smoking, pregnancy, or lactation. The participant demographics were 87.06% Kuwaitis and 12.94% non-Kuwaitis, comprising 12 Egyptians, three Jordanians, two Syrians, two Indians, one Palestinian, one Filipino, and one Bengali. The mean age of the women in the study was 29.19 ± 9.45 years. Participants diagnosed with MetS using the IDF criteria were older than those without MetS (*p* < 0.001), representing 24% of the sample (*n* = 41). Conversely, 18% (*n* = 30) of participants were diagnosed with MetS according to the ATP III criteria. Most women (78.24%) reported having a regular menstrual cycle, and approximately 5.88% were at the menopausal stage. Women with MetS showed a higher tendency to report irregular menses and menopause than those of their healthy counterparts (*p* < 0.05). Additionally, women with MetS exhibited a higher BMI of 28.62 ± 3.80 compared to that of those without MetS (24.16 ± 3.87) (*p* < 0.001). The prevalence and mean cardiovascular risk factors are detailed in Supplementary Table S1. Based on the IDF diagnostic criteria, the most frequent component of MetS was an obesogenic waist at 59.41%, followed by low HDL-C at 37.06%, high FBG level at 22.35%, high TG level at 21.18%, high SBP at 13.53%, and high DBP at 9.41%. Statistically significant differences were observed in the prevalence of all cardiovascular risk factors between participants with and without MetS (*p* < 0.05), except for TC. Mean differences were observed for all cardiovascular risk factors, with women with MetS exhibiting an impaired cardiometabolic profile compared with those without MetS (*p* < 0.05).

**Table 1:** Descriptive statistics for sociodemographic and lifestyle characteristics.

Categorical variables, *N* (%)		Total *N* = 170	With MetS 41 (24)	Without MetS 129 (76)	*P*-value^a^
Nationality	Kuwaiti	148 (87.1)	37 (90.2)	111 (86)	0.49
	Non-Kuwaiti	22 (12.9)	4 (9.8)	18 (14)	
Marital status	Married	75 (44.1)	23 (56.1)	52 (40.3)	0.20
	Single	86 (50.6)	16 (39)	70 (54.3)	
	Divorced	9 (5.3)	2 (4.9)	7 (5.4)	
Educational level	High school or lower	35 (20.6)	8 (19.5)	27 (20.9)	0.84
	College degree or higher	135 (79.4)	33 (80.5)	102 (79.1)	
Employment status	Not employed	78 (45.9)	18 (43.9)	60 (46.5)	0.77
	Employed	92 (54.1)	23 (56.1)	69 (53.5)	
Total family income/year *Missing *n* = 74	<5,000 KD	35 (20.6)	8 (19.5)	27 (20.9)	0.25
	≥5,000 KD	61 (35.9)	19 (46.3)	42 (32.6)	
Regular menses	No	37 (21.8)	17 (41.5)	20 (15.5)	0.0004
	Yes	133 (78.2)	24 (58.5)	109 (84.5)	
Menopause	No	160 (94.1)	34 (82.9)	126 (97.7)	0.0005
	Yes	10 (5.9)	7 (17.1)	3 (2.3)	
Use of hormones or birth control	No	157 (92.3)	39 (95.1)	118 (91.5)	0.44
	Yes	13 (7.7)	2 (4.9)	11 (8.5)	
Family history of	Diabetes	114 (67.1)	29 (70.7)	85 (65.9)	0.57
	Hypertension	105 (61.8)	26 (63.4)	79 (61.2)	0.80
	Heart problems	32 (18.8)	6 (14.6)	26 (20.2)	0.43
Weight Status	Underweight	7 (4.1)	–	7 (5.4)	<0.001
	Normal	85 (50)	3 (7.3)	82 (63.6)	
	Overweight	58 (34.1)	29 (70.7)	29 (22.5)	
	Obese	20 (11.8)	9 (21.9)	11 (8.5)	
Continuous variables, Mean ± SD					
Body mass index, kg/m^2^		25.23 ± 4.29	28.6 2 ± 3.80	24.16 ± 3.87	<0.001
		24.39 [22.39–27.68]	27.44 [26.34–29.70]	23.33 [22.03–25.50]	
Total calories, kcal/day		2718.68 ± 1507.79	2624.45 ± 1715.43	2748.63 ± 1441.63	0.32
		2466.38 [1497.05–3682.73]	2084.18 [1340–3495.99]	2560.74 [1624.94–3681.46]	
Total METs		1119.41 ± 1409.73	936.79 ± 967.27	1177.67 ± 1522.28	0.39
		720.00 [240.00–1680.00]	600.00 [249.00–1380.00]	840.00 [240.00–1680.00]	
Sitting time, hours		6.32 ± 2.04	6.46 ± 2.45	6.28 ± 1.91	0.58
		6.00 [5.00–8.00]	7.00 [5.00–8.00]	6.00 [5.00–8.00]	
Age (years)		29.19 ± 9.45	35.41 ± 12.11	27.21 ± 7.45	<0.001
		27.00 [22.00–34.25]	33.00 [28.00–41.00]	26.00 [21.00–32.00]	

MetS, metabolic syndrome; KD, Kuwaiti Dinar; SD, standard deviation, METs, metabolic equivalents of tasks. Data are expressed as frequency and percentages and mean and standard deviation for categorical and continuous variables, respectively. ^a^Significance level using the Mann–Whitney *U*-test and chi-square test for continuous and categorical variables, respectively.

### 3.2 Serum magnesium, dietary magnesium, and fiber intakes

Serum magnesium concentrations, dietary magnesium, and fiber intake levels are presented in Table 2. Using the standard cutoff point for defining hypomagnesemia, low serum magnesium levels were observed in 29.41% of the total sample, with a significantly higher prevalence of 75.61% among those with MetS compared to 14.73% among those without MetS (*p* < 0.001). This deficiency status increased to 68.8% in the total sample using the lower cutoff value, with 95.1% in those with MetS compared to 60.5% in those without MetS (*p* < 0.001). The median magnesium intake for all women was 508.23 mg/day (IQR: 322.23–839.43), with 18.8% below EAR (24.1% below RDA) for age. Dietary magnesium intake was lower among women with MetS: 237.33 (162.87–351.10), and 58.5% of women with MetS were below the EAR, while 65.9% were below the RDA for their age group. The median intake among women without MetS was 640.06 (410.59–899.76), with 6.2 and 10.9% falling below the EAR and RDA for age, respectively. Using the body weight corrected EAR resulted in magnesium inadequacy rates similar to those from the RDA, with 23.5% for the total sample, 65% among women with MetS, and 10.1% among those without MetS. The median fiber intake for all women was 32.12 g/day (IQR: 20.64–57.67), with 25.3% falling below the dietary reference intake (DRI) for age. Women with MetS had lower fiber intake compared to women without MetS (16.81 [11.24–25.23] vs. 37.86 [27.26–64.59] g/day, *p* < 0.001); 65.85% of women with MetS fell below the AI for age compared to 12.40% of those without MetS (*p* < 0.001).

**Table 2:** Means and standard deviations of dietary magnesium and fiber intakes and distribution (%) of adequacy of intake.

	Total	With MetS	Without MetS	*P*-value^a^
Serum Mg, mmol/L	0.804 ± 0.119	0.691 ± 0.073	0.840 ± 0.10	<0.001
	0.790 [0.728–0.870]	0.690 [0.625–0.735]	0.810 [0.760–0.930]	<0.001
% sMg < 0.74 mmol/L	29.4	75.6	14.7	<0.001
% sMg < 0.85 mmol/L	68.8	95.1	60.5	<0.001
Dietary Mg, mg	598.45 ± 371.38	283.9 ± 170.6	698.4 ± 362.3	<0.001
	508.23 [322.24–839.43]	237.33 [162.87–351.10]	640.06 [410.59–899.76]	<0.001
Mg (mg)/1,000 kcal	286.86 ± 294.80	142.66 ± 15.19	332.6 ± 28.26	<0.001
	226.87 [137.89–312.75]	116.49 [62.31–205.98]	246.05 [190.93–374.04]	<0.001
% ≥ EAR	81.2	41.5	93.8	<0.001
% Below EAR	18.8	58.5	6.2	<0.001
% ≥ RDA	75.9	34.1	89.1	<0.001
% Below RDA	24.1	65.9	10.9	<0.001
% ≥ EAR BW corrected	76.5	34.1	89.9	<0.001
% Below EAR BW corrected	23.5	65.9	10.1	<0.001
Dietary Fiber, g	41.60 ± 30.31	20.4 ± 15.1	48.4 ± 30.8	<0.001
	32.12 [20.64–57.67]	16.81 [11.24–25.23]	37.86 [27.26–64.59]	<0.001
Fiber (g)/1,000 kcal	20.02 ± 23.71	10.24 ± 8.14	23.13 ± 2.30	<0.001
	14.41 [8.13–23.46]	7.12 [3.65–15.52]	15.91 [10.55–25.84]	<0.001
% ≥ AI	74.7	34.1	87.6	<0.001
% below AI	25.3	65.9	12.4	<0.001

DRI, dietary reference intake; RDA, recommended dietary allowance; mg, milligram; g, grams; MetS, metabolic syndrome; EAR, estimated average requirement; AI, adequate intake; BW, body weight. Categorical data are expressed as frequency (N) and percentage (%); continuous data are expressed as mean ± standard deviation and median [Q25th–Q75th]. ^a^The significance level for continuous variables was determined using the Mann–Whitney *U* test and for categorical variables using the chi-square test. Dietary magnesium adequacy was evaluated according to (1) the EAR for magnesium intake, 255 mg for 19–30-year-old females and 265 mg for ≥31-year-old females; (2) RDAs for magnesium intake, 310 mg for 19–30-year-old females and 320 mg for ≥31-year-old females; and (3) body weight-adjusted EAR calculated based on the population-based average weight for Kuwaiti adult women (73 kg) for magnesium intake 305.14 and 316.82 mg, respectively. The adequacy of dietary fiber was evaluated according to AI: 25 g for females aged 19–50 years and 21 g for those aged 51 years and above.

### 3.3 Contribution of dietary magnesium and fiber per food group

Table 3 illustrates the mean contribution of dietary magnesium as total mg/day, mg/1000 kcal, and % per food group from the total dietary magnesium intake. The vegetable group was the most significant contributor to dietary magnesium intake (33%), followed by the meat group, including mixed dishes (16%), fruit group (13%), bread (13%), and nuts (11%). The beans and milk groups contributed the least to the total magnesium intake. Individuals with MetS exhibited a significantly lower magnesium intake (mg/day and mg/1000 Kcals) across all food groups (all *p*-values <0.001) compared to those without MetS. Notably, significant differences in the food group contributions to total dietary magnesium (% of total mg/day) were observed in the bread group (15% in those with MetS vs. 12% in those without MetS) and the nuts group (7% in those with MetS vs. 12% in those without MetS). Table 4 illustrates the mean contribution of dietary fiber as total g/day, g/1000 kcal, and % per food group from the total dietary fiber intake. The vegetable (32%), fruit (27%), and bread (16%) groups were the highest contributors to dietary fiber. Conversely, the meat and mixed dishes group contributed the least (2%). Women without MetS had a higher fiber intake in all food groups (all *p*-values <0.001). However, significant differences in percentage contribution were noted only in the nuts group (10% in those without MetS vs. 6% in those with MetS) and bread group (20% in those with MetS vs. 15% in those without MetS) (all *p*-values <0.05).

**Table 3:** Contribution of dietary magnesium as total mg/day, mg/1000 kcals, and % per food group from the total dietary magnesium intake.

Food groups		Total	With MetS	Without MetS	*P*-value^a^
Vegetable	Total mg/day	208.15 ± 137.28	97.77 ± 76.92	229.21 ± 137.03	
		177.68 [94.24–308.33]	67.79 [48.99–117.15]	209.46 [111.20–347.33]	<0.001
	mg/1000 kcal	48.14 ± 56.19	23.27 ± 20.52	61.57 ± 119.19	
		28.12 [19.29–47.04]	21.35 [10.18–29.68]	30.47 [19.81–59.03]	<0.001
	% of Total mg/day	32.99 ± 12.38	33.18 ± 13.76	32.93 ± 12.33	
		34.26 [23.90–40.85]	31.42 [24.18–43.90]	34.25 [23.68–40.54]	0.938
Meat and mixed dishes^b^	Total mg/day	95.14 ± 70.86	43.58 ± 30.71	107.23 ± 72.91	
		73.73 [44.89–126.94]	36.50 [22.56–63.61]	96.75 [55.77–135.97]	<0.001
	mg/1000 kcal	289.71 ± 79.86	102.40 ± 73.94	320.46 ± 1002.20	
		128.65 [88.20–179.47]	99.35 [39.34–141.13]	137.09 [108.29–233.95]	<0.001
	% of Total mg/day	15.84 ± 7.76	17.60 ± 10.63	16.02 ± 7.66	
		15.37 [10.19–21.09]	16.14 [8.14–25.04]	16.06 [10.40–20.92]	0.596
Fruit	Total mg/day	91.37 ± 93.07	40.45 ± 37.82	101.91 ± 97.21	
		57.74 [36.76–113.03]	29.06 [16.29–54.24]	70.45 [39.76–136.28]	<0.001
	mg/1000 kcal	675.54 ± 1807.42	284.14 ± 225	765.84. ± 1956.93	
		243.83 [164.74–480.33]	221.89 [37.09–307.10]	255.32 [196.40–480.33]	0.005
	% of Total mg/day	13.41 ± 7.54	13.16 ± 9.1	13.28 ± 7.40	
		12.14 [8.16–17.08]	10.93 [6.59–19.13]	12.14 [8.02–17.37]	0.689
Bread	Total mg/day	71.64 ± 62.52	38.30 ± 27.63	78.95 ± 65.85	
		46.88 [26.96–98.83]	31.24 [18.24–45.76]	62.35 [28.70–104.45]	<0.001
	mg/1000 kcal	222.37 ± 293.82	141.09 ± 185.10	237.70 ± 308.77	
		125.94 [59.13–297.49]	70.69 [37.39–139.94]	145.35 [65.82–303.04]	0.005
	% of Total mg/day	12.16 ± 9.65	15.42 ± 11.77	11.63 ± 8.71	
		9.67 [6.17–14.92]	11.71 [7.62–18.60]	9.51 [6.13–14.31]	0.030
Nuts	Total mg/day	77.24 ± 84.85	24.48 ± 25.60	86.04 ± 92.91	
		43.75 [23.62–110.98]	19.24 [5.71–32.36]	55.95 [24.91–120.78]	<0.001
	mg/1000 kcal	769.83 ± 1628.78	195.48 ± 215.94	904.02 ± 1772.40	
		297.97 [108.69–640.35]	126.43 [24.61–304.57]	317.41 [142.58–830.89]	<0.001
	% of Total mg/day	11.43 ± 9.05	7.37 ± 5.08	12.15 ± 10.26	
		9.38 [5.62–16.02]	7.96 [3.03–10.68]	9.47 [4.95–17.53]	0.017
Bean	Total mg/day	55.22 ± 53.64	24.27 ± 37.91	59.03 ± 53.36	
		37.72 [132.25–520.09]	16.67 [6.39–27.49]	38.96 [23.51–78.01]	<0.001
	mg/1000 kcal	609.34 ± 1262.16	232.40 ± 327.03	673.59 ± 1334.68	
		231.41 [132.25–520.09]	191.71 [34.47–272.81]	238.05 [163.47–628.95]	0.002
	% of Total mg/day	8.69 ± 5.96	7.79 ± 6.09	8.4 ± 5.76	
		7.76 [3.77–11.82]	7.39 [3.16–9.78]	7.18 [3.76–11.74]	0.440
Milk	Total mg/day	32.63 ± 27.29	15.1 ± 14.5	35.97 ± 27.24	
		25.24 [12.38–46.17]	10.00 [5.09–19.99]	27.93 [16.40–51.91]	<0.001
	mg/1000 kcal	202.15 ± 429.64	120.91 ± 165.64	212.77 ± 447.59	
		121.62 [63.72–208.45]	85.10 [34.06–125.28]	128.28 [72.43–218.78]	0.001
	% of Total mg/day	55.48 ± 3.87	5.49 ± 3.86	5.57 ± 4.13	
		4.84 [2.52–7.33]	4.94 [2.45–7.06]	4.85 [2.63–7.24]	0.993

MetS, metabolic syndrome. Data are presented as the mean and standard deviation and median (Q25th–Q75th). ^a^Significance level for independent Mann–Whitney *U*-test; ^b^the mixed dishes consumed in Kuwait contain a mixture of any of the following: meat, beans, grains, nuts, and vegetables.

**Table 4:** Contribution of dietary fiber as total g/day, g/1,000 kcals, and % per food group from the total dietary fiber intake.

Food groups		Total *N* = 170	With MetS *N* = 41 (24%)	Without MetS *N* = 129 (76%)	*P*-value^a^
Vegetable	Total g/day	13.49 ± 9.48	7.27 ± 5.90	15.20 ± 9.58	<0.001
		11.32 [5.06–21.12]	5.23 [3.46–8.16]	13.73 [7.69–23.09]	
	g/1000 kcal	4.79 ± 5.61	1.99 ± 1.35	5.55 ± 6.08	<0.001
		2.81 [1.92–4.69]	1.98 [0.84–2.83]	3.14 [2.07–7.50]	
	% of Total g/day	31.78 ± 12.86	32.72 ± 14.27	31.52 ± 12.50	0.42
		31.51 [22.02–39.57]	30.67 [21/19–39.40]	31.84 [22.10–39.84]	
Meat and mixed dishes^b^	Total g/day	0.72 ± 0.60	0.42 ± 0.37	0.80 ± 0.62	<0.001
		0.57 [0.22–1.05]	0.29 [0.19–0.57]	0.65 [0.27–1.26]	
	g/1000 kcal	0.24 ± 1.05	0.07 ± 0.06	0.29 ± 1.18	<0.001
		0.09 [0.05–0.16]	0.05 [0.03–0.10]	0.10 [0.05–0.18]	
	% of Total g/day	2.03 ± 1.69	2.54 ± 2.19	1.88 ± 1.50	0.55
		1.73 [0.70–3.06]	2.17 [0.71–3.86]	1.72 [0.69–2.52]	
Fruit	Total g/day	13.65 ± 15.26	5.73 ± 4.60	15.83 ± 16.42	<0.001
		8.50 [5.05–16.88]	4.47 [3.16–6.65]	9.05 [6.11–19.52]	
	g/1000 kcal	10.11 ± 28.47	3.63 ± 5.27	11.89 ± 31.83	<0.001
		3.71 [2.46–6.90]	2.73 [0.45–4.60]	3.88 [2.56–8.21]	
	% of Total g/day	28.07 ± 14.03	25.75 ± 13.39	28.71 ± 14.18	0.24
		26.02 [18.80–35.97]	24.22 [17.59–33.85]	26.63 [19.57–36.28]	
Bread	Total g/day	6.04 ± 5.16	3.67 ± 2.45	6.69 ± 5.52	<0.001
		4.19 [2.48–8.41]	3.17 [1.70–4.41]	5.32 [2.56–8.84]	
	g/1000 kcal	1.89 ± 2.45	1.34 ± 1.61	2.04 ± 2.62	0.003
		1.20 [0.50–2.44]	0.71 [0.36–1.70]	1.32 [0.54–2.59]	
	% of Total g/day	15.49 ± 11.19	19.32 ± 12.11	14.44 ± 10.74	0.02
		12.74 [8.19–20.27]	18.99 [10.05–27.41]	11.91 [7.80–17.07]	
Nuts	Total g/day	3.85 ± 4.19	1.53 ± 1.59	4.49 ± 4.46	<0.001
		2.25 [1.07–5.68]	1.18 [0.41–1.95]	3.10 [1.41–6.71]	
	g/1000 kcals	3.84 ± 8.10	1.07 ± 1.24	4.61 ± 8.98	<0.001
		1.54 [0.53–3.28]	0.46 [0.13–1.63]	1.60 [0.87–4.15]	
	% of Total g/day	9.01 ± 7.73	6.52 ± 4.57	9.69 ± 8.28	0.011
		7.14 [3.96–12.02]	5.53 [3.49–8.98]	7.49 [4.02–12.74]	
Bean	Total g/day	6.08 ± 7.04	3.36 ± 5.54	6.83 ± 7.24	<0.001
		3.33 [1.46–8.12]	2.44 [0.76–3.33]	4.27 [1.92–9.35]	
	g/1000 kcal	5.56 ± 9.33	1.83 ± 1.57	6.58 ± 10.28	0.002
		2.36 [0.89–0.04]	1.51 [0.46–3.27]	2.57 [1.07–7.78]	
	% of Total g/day	13.63 ± 10.47	13.16 ± 9.66	13.76 ± 10.72	0.76
		10.67 [5.27–19.12]	12.09 [5.16–17.77]	9.73 [5.18–20.67]	

MetS, metabolic syndrome. Data are presented as mean and standard deviation. ^a^Significance level using the independent Mann–Whitney *U* test; ^b^the mixed dishes consumed in Kuwait contain a mixture of any of the following: meat, beans, grains, nuts, and vegetables.

### 3.4 Associations of dietary magnesium and fiber with serum magnesium levels

Table 5 shows the partial Spearman’s correlation between the serum and dietary magnesium levels. A strong correlation was observed between the two variables (*r* = 0.95, *p* < 0.001), and adjustments for total caloric intake, age, and other covariates slightly attenuated the magnitude of the associations (Table 5). Multivariate linear regression analysis also revealed significant associations among serum magnesium, dietary magnesium, and fiber intake (Table 6). However, the beta coefficients were relatively small. Therefore, we created models with 80-mg increments of dietary magnesium (equivalent to 30 g almonds) and 16-g increments of fiber (equivalent to a cup of lentils) to obtain better estimates. Both dietary magnesium and fiber were significantly associated with serum magnesium, with associations exceeding 94% (Table 6).

**Table 5:** Partial Spearman’s correlation coefficients for the association between dietary magnesium and serum magnesium (*N* = 170).

	Model 1	Model 2	Model 3	Model 4
Serum magnesium – dietary magnesium (mg/d)	0.957 (0.942–0.968) *P* < 0.001	0.908, *P* < 0.001	0.899, *P* < 0.001	0.899, *P* < 0.001
Serum magnesium – dietary fiber (g/d)	0.917 (0.888–0.938), *P* < 0.001	0.854, *P* < 0.001	0.843, *P* < 0.001	0.842, *P* < 0.001
Dietary magnesium – dietary fiber	0.964 (0.951–0.974) *p* < 0.001	0.953, *p* < 0.001	0.951, *p* < 0.001	0.950, *p* < 0.001

Data are presented as correlation coefficients (*P*-values). Model 1 was adjusted for total caloric intake; Model 2 was adjusted for total caloric intake and age; Model 3 was adjusted for total caloric intake, age, and body mass index; and Model 4 was adjusted for total caloric intake, age, body mass index, and regular menses.

**Table 6:** Beta (β) estimates and 95% confidence intervals (CIs) for the association between serum magnesium (dependent variable) (*N* = 170) and dietary magnesium and fiber intakes (independent variables).

	Dietary magnesium			Dietary fiber		
	Total Mg intake (mg)		For every 80-mg increase in dietary magnesium	Total fiber intake (g)		For every 16-g increase in dietary fiber
	Β (95% CI)	*P*-value		Β (95% CI)	*P*-value	
Model 1	0.00029 (0.00027, 0.00031)	<0.001	0.02346 (0.02190, 0.02503)	0.00339 (0.00309, 0.00368)	<0.001	0.05418 (0.04937 0.05901)
Model 2	0.00029 (0.00027, 0.00031)	<0.001	0.02313 (0.02152, 0.02474)	0.00330 (0.00300, 0.00360)	<0.001	0.05288 (0.04801, 0.05775)
Model 3	0.00029 (0.00027, 0.00031)	<0.001	0.02318 (0.02155, 0.02482)	0.00330 (0.00299, 0.00361)	<0.001	0.05277 (0.04784, 0.05770)
Model 4	0.00029 (0.00027, 0.00031)	<0.001	0.02314 (0.02149, 0.02479)	0.00329 (0.00298, 0.00360)	<0.001	0.05258 (0.04762, 0.05755)
Model 5	0.00029 (0.00026, 0.00031)	<0.001	0.02282 (0.02110, 0.02454)	0.00319 (0.00288, 0.00351)	<0.001	0.05108 (0.04603, 0.05613)

Model 1, unadjusted; Model 2, Model 1 plus age; Model 3, Model 2 plus total caloric intake; Model 4, Model 3 plus regular menses; Model 5, Model 4 plus body mass index.

Table 7 presents Cohen’s kappa coefficients assessing the agreement between dietary magnesium inadequacy (based on EAR and RDA) and serum magnesium deficiency (using cutoffs of 0.74 and 0.85 mmol/L). There was strong agreement between dietary magnesium inadequacy based on EAR and serum magnesium deficiency using the 0.74 mmol/L cutoff (*K* = 0.715 [0.614, 0.816], *p*-value <0.001). High sensitivity (87%) and specificity (64%) were observed between the two measures. Conversely, agreement was poor when using the lower 0.85 mmol/L cutoff (*K* = 0.190 [0.131, 0.249], *p* < 0.001). Using RDA levels for dietary magnesium with both serum magnesium cutoffs improved agreement (Table 7).

**Table 7:** Cohen’s kappa coefficient for the agreement between magnesium deficiency based on dietary magnesium and serum magnesium.

	Serum Mg, mmol/L					
Dietary Mg	0.74	<0.74	Total	Cohen’s kappa coefficient	95% confidence intervals	*P*-value
EAR	120 (87%)^a^	18	138	0.715	(0.614, 0.816)	<0.001
<EAR	0	32 (64%)^b^	32			
Total	120	50	170			
	**0.74**	**<0.74**	Total			
RDA	119 (90.8%)^a^	12	120	0.803	(0.702, 0.904)	<0.001
<RDA	1	38 (76%)^b^	50			
Total	120	50	170			
	**8.5**	**<8.5**				
EAR	53 (38.4%)^a^	85	120	0.190	(0.131, 0.249)	<0.001
<EAR	0	32 (27.4%)^b^	50			
Total	53	117	170			
	**8.5**	**<8.5**	Total			
RDA	53 (40.5%)^a^	78	120	0.238	(0.170, 0.306)	<0.001
<RDA	0	39 (33.3%)^b^	50			
Total	53	117	170			

Dietary magnesium adequacy was evaluated according to (1) the EAR for magnesium intake: 255 mg for females aged 19–30 years and 265 mg for those aged 31 years and above; and (2) RDAs for magnesium intake: 310 mg for females aged 19–30 years and 320 mg for those aged 31 years and above. ^a^% sensitivity and ^b^% specificity. EAR, estimated average requirement; RDA; recommended dietary allowance. The MOH population-specific reference ranges defined hypomagnesemia as a serum Mg level of < 0.74 mmol/L (5), and the newly proposed lower reference cut-off value of < 0.85 mmol/L suggested by Rosanoff et al. (38) to identify individuals at risk of chronic latent magnesium deficit.

### 3.5 MetS and its components with dietary magnesium and fiber

As shown in Table 8, dietary magnesium and fiber exhibited strong positive collinearity [*r*(170) = 0.96, *p* < 0.0001]. Additionally, both dietary fiber and magnesium showed strong positive correlations with serum magnesium levels [*r*(170) = 0.92, *p* < 0.001 and *r*(170) = 0.96, *p* < 0.001, respectively]. Serum magnesium, dietary magnesium, and fiber were significantly associated with cardiometabolic risk factors, except for SBP and DBP (all *p*-values <0.05; Table 8), and the same correlation was observed between dietary magnesium and fiber and cardiometabolic risk factors. Table 9 shows the association between dietary magnesium and dietary fiber and the odds of developing MetS and its components. Across all models, higher dietary magnesium intake was associated with lower odds of developing MetS and its components in the crude and adjusted models (*p* < 0.001), except for SBP and DBP, the significance of which was attenuated after covariate adjustment. Model 4B (Table 9) shows that an increase in magnesium intake by 30 g (equivalent to one serving of almond) was associated with lower odds of developing MetS by 45% (adjusted OR [aOR] = 0.55 [95% CI 0.44, 0.68], *p* < 0.001). Adjusting for age and the significant covariates, including total energy, having regular menses, and BMI (Model 5B, Table 9), did not change the magnitude or significance (OR = 0.56 [95% CI 0.44, 0.72], *p* < 0.001). Similarly, higher dietary fiber intake (g/day) was associated with lower odds of developing MetS and its components in the crude and adjusted models (*p* < 0.001), except for blood pressure. Model 5D (Table 9), which was the fully adjusted model, showed that increasing fiber intake by 16 g, equivalent to a cup of boiled lentils, reduced the odds of developing MetS by 72% (aOR = 0.28 [95% CI 0.16, 0.50], *p* < 0.001). SBP was significantly associated with fiber (g) in the crude mode only (OR = 0.967 [0.943, 0.992], *p* = 0.010) but not after adjustment. DBP was not associated with fiber content in the crude or adjusted models. Both dietary magnesium and fiber inadequacies were strongly associated with 13-fold higher odds of developing MetS (aOR = 13.29 [95% CI 5.03, 35.13], *p* < 0.001 and aOR = 13.06 [95% CI 4.68, 36.45], *p* < 0.001, respectively; Supplementary Table S2). These adjusted models collectively explained 54% of the variation in the risk of MetS.

**Table 8:** Spearman’s correlation coefficients for the associations among dietary magnesium, fiber, serum magnesium, and cardiovascular risk factors (*N* = 170).

Spearman’s Rho	Serum Mg, mg	Dietary Mg, mg	Dietary Fiber, g
Waist circumference, cm	−0.434 (<0.0001)	−0.419 (<0.0001)	−0.353 (<0.0001)
BMI (kg/m^2^)	−0.443 (<0.0001)	−0.429 (<0.0001)	−0.385 (<0.0001)
SBP, mmHg	−0.146 (0.0567)	−0.129 (0.0933)	−0.131 (0.0894)
DBP, mmHg	−0.145 (0.0595)	−0.137 (0.0748)	−0.116 (0.1311)
FBG, mmol/L	−0.593 (<0.0001)	−0.574 (<0.0001)	−0.509 (<0.0001)
TG, mmol/L	−0.663 (<0.0001)	−0.624 (<0.0001)	−0.589 (<0.0001)
HDL-C, mmol/L	0.399 (<0.0001)	0.442 (<0.0001)	0.407 (<0.0001)
LDL-C, mmol/L	−0.300 (<0.0001)	−0.257 (0.0007)	−0.238 (0.0018)
TC, mmol/L	−0.328 (<0.0001)	−0.262 (0.0006)	−0.229 (0.0026)
Serum Mg, mg		0.957 (<0.0001)	0.917 (<0.0001)
Dietary Mg, mg	0.957 (<0.0001)		0.964 (<0.0001)
Total Fiber, g	0.917 (<0.0001)	0.964 (<0.0001)	

BMI, body mass index; SBP, systolic blood pressure; DBP, diastolic blood pressure; FBG, fasting blood glucose; TG, triglyceride; HDL-C, high-density lipoprotein cholesterol; LDL-C, low-density lipoprotein cholesterol; TC, total cholesterol; Mg, magnesium; mg milligram, g, gram. Data are presented as correlation coefficients (*P*-values).

**Table 9:** Multivariate logistic regression models for metabolic syndrome as the outcome variable and dietary magnesium or fiber as exposure variables.

	Dietary magnesium			Dietary fiber		
	A. Mg/day as a continuous variable, OR (95% CI)	*P*-value	B. For every 80-mg increase in dietary magnesium	C. g/day as a continuous variable, OR (95% CI)	*P*-value	D. For every 16-g increase in dietary fiber
Metabolic syndrome						
Model 1	0.992 (0.990, 0.995)	<0.001	0.546 (0.437, 0.682)	0.924 (0.894, 0.955)	<0.001	0.283 (0.168, 0.477)
Model 2	0.993 (0.990, 0.996)	<0.001	0.558 (0.445, 0.699)	0.926 (0.895, 0.958)	<0.001	0.293 (0.170, 0.504)
Model 3	0.993 (0.990, 0.995)	<0.001	0.550 (0.437, 0.692)	0.924 (0.892, 0.958)	<0.001	0.284 (0.162, 0.500)
Model 4	0.993 (0.990, 0.996)	<0.001	0.563 (0.444, 0.715)	0.927 (0.893, 0.962)	<0.001	0.296 (0.163, 0.536)
High waist circumference						
Model 1	0.997 (0.995, 0.998)	<0.001	0.759 (0.689, 0.836)	0.965 (0.951, 0.979)	<0.001	0.562 (0.446, 0.708)
Model 2	0.996 (0.995, 0.998)	<0.001	0.746 (0.664, 0.837)	0.963 (0.947, 0.980)	<0.001	0.549 (0.417, 0.723)
Model 3	0.996 (0.995, 0.998)	<0.001	0.750 (0.667, 0.844)	0.964 (0.948, 0.981)	<0.001	0.560 (0.425, 0.738)
Model 4	0.998 (0.996, 0.999)	0.012	0.819 (0.702, 0.957)	0.978 (0.958, 0.999)	0.044	0.706 (0.503, 0.991)
High systolic blood pressure						
Model 1	0.997 (0.995, 0.999)	0.002	0.786 (0.674, 0.918)	0.967 (0.943, 0.992)	0.010	0.588 (0.393, 0.881)
Model 2	0.998 (0.996, 1.000)	0.078	0.860 (0.727, 1.017)	0.979 (0.953, 1.005)	0.114	0.708 (0.461, 1.087)
Model 3	0.999 (0.996, 1.001)	0.206	0.893 (0.750, 1.064)	0.984 (0.958, 1.012)	0.259	0.778 (0.504, 1.203)
Model 4	0.999 (0.997, 1.001)	0.356	0.919 (0.767, 1.100)	0.987 (0.960, 1.015)	0.369	0.815 (0.522, 1.274)
High diastolic blood pressure						
Model 1	0.997 (0.995, 0.999)	0.015	0.803 (0.673, 0.957)	0.976 (0.950, 1.002)	0.069	0.674 (0.441, 1.031)
Model 2	0.999 (0.997, 1.001)	0.394	0.920 (0.759, 1.115)	0.993 (0.965, 1.022)	0.637	0.896 (0.568, 1.413)
Model 3	0.999 (0.997, 1.002)	0.670	0.957 (0.784, 1.169)	0.999 (0.971, 1.027)	0.928	0.979 (0.622, 1.541)
Model 4	1.000 (0.997, 1.003)	0.956	0.994 (0.806, 1.225)	1.003 (0.973, 1.033)	0.864	1.042 (0.648, 1.677)
High blood glucose levels						
Model 1	0.995 (0.993, 0.997)	<0.001	0.675 (0.573, 0.795)	0.956 (0.934, 0.979)	0.0002	0.488 (0.336, 0.709)
Model 2	0.995 (0.993, 0.998)	<0.001	0.695 (0.590, 0.819)	0.960 (0.938, 0.983)	0.0008	0.523 (0.358, 0.763)
Model 3	0.995 (0.993, 0.997)	<0.001	0.690 (0.582, 0.817)	0.960 (0.937, 0.984)	0.0012	0.525 (0.355, 0.776)
Model 4	0.996 (0.994, 0.998)	0.001	0.712 (0.599, 0.847)	0.965 (0.941, 0.990)	0.006	0.568 (0.380, 0.849)
High triglyceride levels						
Model 1	0.994 (0.991, 0.996)	<0.001	0.611 (0.501, 0.744)	0.922 (0.891, 0.955)	<0.001	0.274 (0.157, 0.480)
Model 2	0.994 (0.992, 0.997)	<0.001	0.628 (0.516, 0.764)	0.925 (0.893, 0.959)	<0.001	0.289 (0.165, 0.509)
Model 3	0.994 (0.991, 0.996)	<0.001	0.603 (0.490, 0.743)	0.919 (0.884, 0.954)	<0.001	0.257 (0.140, 0.471)
Model 4	0.994 (0.991, 0.997)	<0.001	0.619 (0.501, 0.764)	0.921 (0.887, 0.958)	<0.001	0.270 (0.146, 0.500)
Reduced high-density lipoprotein cholesterol levels						
Model 1	0.998 (0.997, 0.999)	<0.001	0.833 (0.761, 0.912)	0.974 (0.959, 0.988)	0.0004	0.651 (0.514, 0.824)
Model 2	0.998 (0.996, 0.999)	<0.001	0.827 (0.753, 0.908)	0.973 (0.958, 0.988)	0.0003	0.644 (0.506, 0.820)
Model 3	0.998 (0.996, 0.999)	0.0001	0.829 (0.753, 0.912)	0.973 (0.959, 0.988)	0.0005	0.650 (0.509, 0.830)
Model 4	0.998 (0.996, 0.999)	0.0002	0.828 (0.750, 0.915)	0.974 (0.959, 0.989)	0.0009	0.655 (0.510, 0.841)

OR, odds ratio; CI, confidence interval. Model 1, unadjusted model; Model 2, Model 1 plus age; Model 3, Model 2 plus regular menses; Model 4, model 3 plus body mass index.

## 4 Discussion

To the best of our knowledge, this is the first study to provide critical insights into the association between dietary magnesium and fiber intake and the prevalence of MetS among apparently healthy women in Kuwait. Notably, this study identified significant differences in dietary intakes and biochemical markers between women with and without MetS. Our results indicate that a higher dietary intake of magnesium and fiber is associated with a lower likelihood of developing MetS and its components.

The high prevalence of hypomagnesemia among women with MetS was significantly correlated with a lower dietary magnesium intake. The strong agreement between dietary magnesium inadequacy and low serum magnesium concentrations (Cohen’s kappa = 0.70) suggests that dietary assessments can reliably reflect magnesium status in the body. In addition, the robust correlation between serum magnesium levels and dietary intake supported the validity of the SFFQ. This correlation mirrors findings from Akizawa et al. (43), who reported a significant correlation between serum and dietary magnesium among Japanese adults (*r* = 0.29). The higher correlation found in our study underscores the SFFQ’s effectiveness, specifically designed to assess dietary magnesium and fiber intakes in Kuwait, including traditional and habitually consumed mixed dishes, modifying portion sizes and using three-dimensional food models with the SFFQ effectively captured participants’ dietary habits, ensuring the accuracy of the dietary intake data.

The study revealed that dietary magnesium and fiber intakes were significantly lower among women with MetS than among those without MetS. Our tool identified inadequacies in magnesium intake, with 18.8% of women in the overall sample falling below the EAR for their age (24.1% below RDA). Furthermore, 58.5% of women with MetS fell below the EAR compared to 6.2% of those without MetS (65.9 and 10.9% below RDA, respectively). When using the body weight-corrected EAR to adjust for increases in body weight above the standard reference body weight of the 1997 DRIs (36), the rates of magnesium inadequacy were similar to those observed when employing the RDA cutoff point: 23.5% of the overall sample fell below the body weight-corrected EAR, with 65.9% of women with MetS and 10.1% of those without MetS falling below this threshold. This deficiency was particularly evident in relation to vegetable, fruit, nut, and whole grain consumption.

Interestingly, the bread group contributed significantly more to magnesium and fiber intake (% of total intake per day) in women with MetS compared to that in those without MetS. Conversely, the bread group’s overall total magnesium and fiber contents were higher among those without MetS. The low magnesium and fiber intake from the bread group of women with MetS can thus be linked with relatively higher consumption of refined white bread with low fiber and magnesium content. High intake of refined bread can lead to a higher glycemic load associated with MetS in patients with type 2 diabetes (44), and reduced fiber intake is linked to metabolic abnormalities (41). Whole-grain breads are rich in both soluble and insoluble fibers. Findings from a previous study indicated that consuming fiber-rich bread may benefit women with MetS (45). Soluble fibers reduce postprandial glycemic excursions by forming gels, increasing gastrointestinal viscosity, and delaying gastric emptying, thus reducing glucose absorption (46). Insoluble fibers, such as resistant starches, lower the glycemic response of foods by enhancing whole-body insulin sensitivity (37, 38).

The vegetable and fruit groups were the highest contributors to dietary fiber, with higher daily consumption per 100 kcal among women without MetS. A dietary pattern rich in fiber has anti-inflammatory properties, which may prevent chronic diseases, including MetS. Some proposed mechanisms include decreased lipid oxidation linked with reduced inflammation and normalization of bowel flora (47–50). In addition, fiber intake reduces energy intake, increases fat excretion, slows down gut motility, increases fecal mass, and combats constipation (51, 52).

In the current study, nuts also significantly contributed to magnesium and fiber intake in the diets of women without MetS. Similar results of an inverse association with MetS have been observed with Mediterranean dietary patterns, including high nut consumption (53). The role of nut consumption in preventing MetS and overweight/obesity has been validated in a meta-analysis of prospective cohort studies. It demonstrated that with every increase of 1 serving per week in nut consumption, the risk decreased by 4% for MetS, 3% for overweight/obesity, and 5% for obesity (54). In addition, pooled data from randomized feeding trials suggest that nut supplementation can lower body weight, BMI, and waist circumference, further supporting the metabolic benefits of nuts (54). Future studies with larger sample sizes may elucidate the effect of nut type on the risk of developing MetS and its components. For example, walnut-enriched diets had statistically significant decreasing effects for TG, TC, and LDL-C concentrations on some inflammatory markers in middle-aged and older adults (55).

In our study, participants had a significantly higher average intake of magnesium (508.22 mg/day, 192% EAR) and fiber (32.12 g/day, 128% AI) than the global average. This intake level may be partly attributed to the use of a detailed SFFQ, which is considered more reliable than the 24-h recall and 1-day meal recording methods used in other studies. This methodology captured all food sources of magnesium and fiber, unlike other FFQs, which may overlook significant sources such as mixed dishes and nuts (41, 43, 56–58). In addition, the high average intake of dietary magnesium and fiber in our study can be attributed to the traditional dietary patterns observed among the women sampled. These patterns are characterized by higher consumption of vegetables, mixed traditional dishes, nuts, and fruits, particularly evident among women without MetS. Interestingly, our data indicated that the meat and mixed dish group ranked as the second-highest contributor to magnesium intake among all women. As noted by Rosanoff (2021) (36), the 1997 RDAs of 310–420 mg magnesium/day fall below the range of measured intakes found in traditional diets, which range from 940 to ≥713 mg magnesium/day. Indeed, women without MetS had a median magnesium intake of 698 mg/day (263% EAR, 225% RDA) vs. 284 mg/day (107% EAR, 91.6% RDA) among those with MetS. Traditional dishes containing lean meat cooked with vegetables, whole grains, and pulses can be used daily to ensure an adequate intake of magnesium. Al Zenki et al. (3) and Keane et al. (59) emphasized the need for public health interventions to address the high prevalence of MetS, addressing both dietary patterns and nutritional therapy.

The inverse relationship between higher dietary magnesium and fiber intake significantly correlated with a lower prevalence of MetS and its components, which is consistent with the results of several previous investigations (33). The consistency of the association with dietary magnesium across studies varies, with two meta-analyses showing a negative association between dietary magnesium and MetS (27, 60). Our findings are supported by two large prospective studies: in the United States of America (USA), among 4,637 young Americans, higher magnesium intake was linked to a 31% reduced risk of developing MetS over 15 years (61). More recently, another prospective study in China involving 6,104 adult participants with a total follow-up of 37,173 person-years also showed that higher dietary magnesium intake was linked to a 16% reduced risk of developing MetS and its components (56). Among 11,686 American women aged 45 years and older, dietary magnesium was associated with a 27% lower MetS risk (22).

In contrast, Bo et al. (41) reported that while fiber intake showed an inverse association with MetS after adjusting for confounders, magnesium intake did not exhibit such an association. They found that low intake of both nutrients increased the risk of MetS by three to four times (41). Ford et al. (58) similarly observed an inverse association between dietary magnesium intake and the prevalence of MetS. However, this association did not persist when they extensively adjusted for demographic factors, lifestyle variables, and dietary confounders (58).

Our study demonstrated that increased dietary magnesium and fiber intakes were associated with reduced odds of developing MetS and its components, except for SBP and DBP. Our logistic regression models, adjusted for various covariates, consistently showed that higher intakes of magnesium and fiber significantly lowered the odds of developing MetS and its components, reinforcing the importance of these nutrients in metabolic health. Our odds ratio estimates for the risk of MetS fall within the range of the multivariable-adjusted hazard ratios reported in the two prospective studies in China (hazard ratio [HR] = 0.84, 95% CI = 0.71–0.99) and the USA (HR = 0.69, 95% CI = 0.52–0.91) (52). Among Saudi and Italian adults, the risk of MetS increased by 2.7 times (OR = 2.7, 95% CI = 1.0–7.2) and 3.26 times (OR = 3.26, 95% CI = 2.41–4.41), respectively, in the lowest quartile of dietary magnesium intake (41, 62). In all the studies where the investigators adjusted for dietary fiber, the association between dietary magnesium and MetS disappeared due to their high intercorrelation (*r* ≥ 0.81) and their frequent coexistence in the same foods (41, 63). With this knowledge of multicollinearity, we constructed separate models for dietary magnesium and fiber and identified the foods that contributed to their levels. Our data showed a strong and significant reduction in MetS risk for both dietary magnesium and fiber with similar strengths and magnitudes when the variables were treated as continuous variables (aOR = 0.993 [0.990–0.996, *p* < 0.001] and aOR = 0.927 [0.893–0.9620, *p* < 0.001] for dietary magnesium and fiber, respectively). However, fiber showed a more pronounced effect when evaluating the impact of dietary magnesium and fiber increments. Specifically, every 16 g increase in dietary fiber content (equivalent to a cup of lentils) demonstrated a more substantial effect than every 80 mg increase in dietary magnesium content (equivalent to 30 g of almonds). These analyses showed that there was a 70% vs. 44% reduction in MetS, 29% vs. 18% in abdominal obesity, 43% vs. 29% in hyperglycemia, 73% vs. 38% in hypertriglyceridemia, and 35% vs. 17% in low HDL in the fully adjusted models. Previous studies had reported similar protective effects of dietary magnesium, with a reduction in central obesity by 18%, elevated TG levels by 41%, decreased HDL-C levels by 23%, elevated glucose levels by 42%, and blood pressure by 20% (56, 64). A few studies on the dose–response relationship between dietary magnesium and its components showed that an increase of 150 mg/day of dietary magnesium decreased the risk of MetS by 12% (risk ratio [RR] = 0.88, 95% CI = 0.84–0.93) (50). Based on a meta-analysis of prospective studies, an increment of 100 mg/day in dietary magnesium was associated with a 16% reduction in the risk of type 2 diabetes (RR: 0.84; 95% CI: 0.80–0.88) (65), and a 5% reduction in the risk of hypertension (RR = 0.95; 95% CI: 0.90, 1.00) (66).

Research has consistently suggested a potential link between dietary magnesium and hypertension (67, 68). However, our study showed no association between dietary magnesium or fiber intake and hypertension. Similarly, Peacock et al. (69) and Burgess et al. (70) found no association between dietary magnesium intake and the incidence of hypertension. McCarron (71) suggested that a reduced consumption of calcium, rather than magnesium, is more consistently associated with hypertension. Not accounting for important dietary factors such as calcium, sodium, potassium, cholesterol, and saturated fat intake could weaken any observed inverse association between blood pressure measures and dietary magnesium if such a relationship exists. These factors are critical for blood pressure regulation and cardiovascular health (59). Additionally, the prevalence of hypertension in our sample was low, at 13.5% for SBP and 9.4% for DBP. The small sample size in our study may have been a major limitation in detecting significant correlations with blood pressure.

Sociodemographic factors, such as age and menstrual status, were significantly associated with the prevalence of MetS. Women diagnosed with MetS were generally older and more likely to report irregular menses or menopause. These findings suggest that age-related hormonal changes contribute to the development of MetS. Reduced estrogen levels and alterations in the testosterone-to-estrogen ratio during the transition to menopause significantly increase the risk of cardiovascular disease through changes in lipid metabolism (72) and the development of obesity and insulin resistance (73). Insulin resistance is linked to the increased accumulation of central adiposity and ectopic fat infiltration in the skeletal muscle and liver (74). Decreased insulin activity and secretion can be attributed to increased fat mass, reduced muscle mass, and general endocrine changes associated with aging (74).

Furthermore, MetS is associated with age-related conditions such as frailty, cognitive decline, and impaired cardiovascular autonomic control (75). Lifestyle modifications, including weight loss and physical activity, prevent MetS in older individuals (76). During pregnancy, women experience gestational diabetes, high blood pressure, and excessive weight gain, which can lead to long-term health problems for the mother, such as postpartum weight retention, obesity, postpartum depression, and MetS (77). This study highlights the high prevalence of MetS among women, influenced by physiological changes during pregnancy and menopause that affect metabolic health (78). Collectively, these findings emphasize the need for early screening and lifestyle interventions tailored to women’s health.

### 4.1 Strengths and limitations

The strengths of this study include the comprehensive dietary assessment using a culturally adapted SFFQ and rigorous biochemical measurements conducted in well-equipped laboratories. Despite its strengths, the study’s limitations include its cross-sectional design, which cannot establish causality, and its focus on a relatively small sample from two governorates, which limits its representativeness. Future longitudinal studies are needed to establish causal relationships between dietary intake and MetS. Additionally, excluding men limits the generalizability of the findings to the entire population. Other limitations include the omission of several potential confounders, such as other dietary components, including calcium and phosphorus, which may interact with dietary magnesium. We also did not include water consumption data; however, the magnesium intake from drinking water was negligible compared to the total magnesium intake from the diet (79).

### 4.2 Public health implications

The findings of this study have important implications for public health strategies aimed at reducing the burden of MetS in Kuwait. Interventions promoting the consumption of magnesium- and fiber-rich foods, such as vegetables, nuts, and whole grains, should be prioritized. Moreover, regular dietary assessments in clinical practice could help identify individuals at risk of nutrient deficiencies and associated metabolic disorders. These findings indicate that while dietary interventions can effectively mitigate several risk factors for MetS, additional strategies may be necessary to address hypertension in women.

## 5 Conclusion

In conclusion, this comprehensive study provides essential insights into the protective effects of dietary magnesium and fiber against MetS, highlighting the importance of dietary management in public health strategies, particularly for women in Kuwait. Research on the impact of dietary and lifestyle factors on MetS has identified several critical areas for future research. Cao et al. (80) and Roos et al. (81) highlighted the need for more comprehensive studies considering various dietary and lifestyle factors, including physical activity, smoking, sleep quality, working conditions, and eating patterns. Iqbal et al. (82) emphasized the importance of considering sociodemographic and lifestyle factors and dietary habits in different ethnic groups. Martínez-González and Martín-Calvo (53) and Keane et al. (59) suggested that future research should focus on the overall dietary patterns and the potential role of functional foods or bioactive components in improving MetS symptoms. Phillips et al. (83) and Calton et al. (84) underscored the need for a personalized nutrition approach that considers gene-nutrient interactions and the potential protective effects of specific dietary patterns. This study highlights the critical role of dietary magnesium and fiber in mitigating the risk of MetS among Kuwaiti women. The strong association between these nutrients and metabolic health underscores the need for dietary interventions and public health policies to promote the intake of magnesium- and fiber-rich foods. Addressing dietary inadequacies through targeted nutritional strategies could significantly reduce the prevalence of MetS and improve the overall metabolic health in this population.

## Data Availability

The raw data supporting the conclusions of this article will be made available by the authors, without undue reservation.
