# Supplementary material for: Low dietary magnesium and fiber intakes among women with metabolic syndrome in Kuwait
Source: Front Nutr. 2024 Oct 16;11:1451220. doi: 10.3389/fnut.2024.1451220 (PMC11521934; doi:10.3389/fnut.2024.1451220)
Supplement: Supplementary file 1 [file Data_Sheet_1.docx]

Supplementary Material

# Supplementary Figures and Tables

## Supplementary Figure


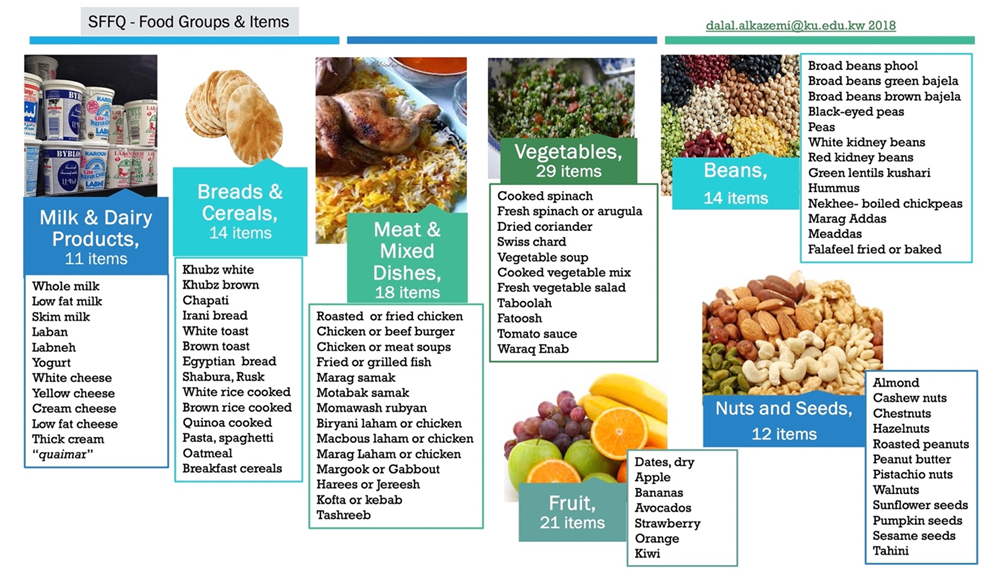


**Supplementary Figure 1.** Examples of food items included in the food frequency questionnaire

## Supplementary Tables

**Supplementary Table 1.** Distributions of cardiovascular risk factors (%) and their mean concentrations

|  | Total | With MetS | Without MetS | P-value ^a^ |
| --- | --- | --- | --- | --- |
| N | 170 | 41 | 129 |  |
| Waist circumference, cm | 84.34$\pm$ 9.42 | 91.99 $\pm$ 9.01 | 81.91 $\pm$ 8.19 | < 0.001 |
|  | 84.00 [78.00–89.00] | 89.00 [86.00–94.50] | 79.00 [77.00–86.75] |  |
| % Obesogenic Waist, ≥ 80 cm | 59.4 | 100 | 46.5 | < 0.001 |
| SBP, mmHg | 120.72$\pm$ 8.35 | 126.02 $\pm$ 10.05 | 119.03 $\pm$ 6.97 | 0.0003 |
|  | 120.00 [118.00–122.00] | 122.00 [120.00–134.00] | 120.00 [117.00–122.00] |  |
| % SBP ≥ 130mmHg | 13.5 | 36.6 | 6.2 | < 0.001 |
| DBP, mmHg | 79.46 $\pm$ 4.13 | 81.71 $\pm$ 4.73 | 78.74 $\pm$ 3.66 | < 0.001 |
|  | 80.00 [78.00–82.00] | 82.00 [79.00–85.00] | 79.00 [77.00–81.00] |  |
| % DBP ≥ 85 mmHg | 9.4 | 26.8 | 3.9 | < 0.001 |
| FBG, mmol/L | 5.15 $\pm$ 0.89 | 6.10 $\pm$ 1.09 | 4.86 $\pm$ 0.56 | < 0.001 |
|  | 5.00 [4.60–5.51] | 5.80 [5.51–6.47] | 4.82 [4.54–5.14] |  |
| % FBG ≥ 5.60 mmol/L | 22.4 | 73.2 | 6.2 | < 0.001 |
| TG, mmol/L | 1.23 $\pm$ 0.83 | 2.12 $\pm$ 1.02 | 0.95 $\pm$ 0.52 | < 0.001 |
|  | 0.99 [0.70–1.55] | 1.96 [1.50–2.86] | 0.86 [0.63–1.10] |  |
| TG ≥ 1.70 mmol/L | 21.2 | 70.7 | 5.4 | < 0.001 |
| HDL-C, mmol/L | 1.42 $\pm$ 0.32 | 1.09$\pm$ 0.28 | 1.53 $\pm$ 0.41 | < 0.001 |
|  | 1.40[1.14–1.58] | 1.06 [0.87–1.25] | 1.45[1.29–1.71] |  |
| HDL-C < 1.29 mmol/L | 37.1 | 78.1 | 31 24 | < 0.001 |
| LDL-C, mmol/L | 2.73$\pm$ 1.01 | 3.08$\pm$ 1.42 | 2.52 $\pm$ 0.80 | 0.0078 |
|  | 2.51 [1.98–3.06] | 2.77 [2.28–3.22] | 2.47[1.90–2.86] |  |
| % LDL-C > 4.12 mmol/L | 7.1 | 17.1 | 3.9 | 0.004 |
| TC, mmol/L | 4.63$\pm$ 1.22 | 5.11$\pm$ 1.5 | 4.47 $\pm$ 1.06 | 0.0031 |
|  | 4.45 [3.82–5.19] | 4.95 [4.22–5.62] | 4.26 [3.76–5.10] |  |
| % TC > 5.17 mmol/L | 26.5 | 36.6 | 23.3 | 0.09 |

MetS, metabolic syndrome; cm, centimeter; SBP, systolic blood pressure; DBP, diastolic blood pressure; FBG, fasting blood glucose; TG, triglyceride; TC, total cholesterol; LDL-C, low-density lipoprotein cholesterol; HDL-C, high-density lipoprotein cholesterol; Mg, magnesium; mg, milligram. ^a^ Significance level using the independent Mann–Whitney U test

**Supplementary Table 2a. Multivariate Logistic Regression for the association between the odds of MetS and Mg Inadequacy.**

| Variables in the Equation | B | S.E. | Wald | df | Sig. | Exp(B) | 95% CI for EXP(B) | |
| --- | --- | --- | --- | --- | --- | --- | --- | --- |
|  |  |  |  |  |  |  | Lower | Upper |
| Mg inadequacy, EAR | 2.879 | 0.544 | 28.033 | 1 | < 0.001 | 17.804 | 6.132 | 51.692 |
| BMI (m^2^/kg) | 0.229 | 0.059 | 15.115 | 1 | < 0.001 | 1.257 | 1.120 | 1.411 |
| Age (years) | 0.042 | 0.028 | 2.259 | 1 | 0.133 | 1.043 | 0.987 | 1.103 |
| Do you have regular periods? Yes = 1 | 0.350 | 0.597 | 0.345 | 1 | 0.557 | 1.420 | 0.441 | 4.574 |
| Constant | -9.736 | 1.770 | 30.264 | 1 | < 0.001 | 0.000 |  |  |
| a. Variable(s) entered in step 1: Mg inadequacy, BMI (m^2^/kg), age (years), do you have regular periods? Yes=1. Nagelkerke R^2^ = 550 | | | | | | | | |

**Supplementary Table 2b. Multivariate Logistic Regression for the association between the odds of MetS and Fiber Inadequacy.**

| Variables in the Equation | B | S.E. | Wald | df | Sig. | Exp(B) | 95% CI for EXP(B) | |
| --- | --- | --- | --- | --- | --- | --- | --- | --- |
|  |  |  |  |  |  |  | Lower | Upper |
| Fiber inadequacy, AI | 2.711 | 0.511 | 28.174 | 1 | < 0.001 | 15.045 | 5.529 | 40.940 |
| BMI (m^2^/kg) | 0.226 | 0.059 | 14.920 | 1 | < 0.001 | 1.254 | 1.118 | 1.406 |
| Age (years) | 0.064 | 0.027 | 5.396 | 1 | 0.020 | 1.066 | 1.010 | 1.124 |
| Do you have regular periods? Yes = 1 | 0.287 | 0.596 | .232 | 1 | 0.630 | 1.333 | 0.414 | 4.286 |
| Constant | -10.517 | 1.829 | 33.066 | 1 | < 0.001 | 0.000 |  |  |
| a. Variable(s) entered in step 1: Fiber inadequacy, BMI (m^2^/kg), age (years), do you have regular periods? Yes=1. Nagelkerke R^2^ = 555 | | | | | | | | |

B, regression Coefficient; S.E., standard error; Wald, Wald chi-square test statistic; df, degrees of freedom; Sig., significance (P-value); Exp(B), exponentiation of the B coefficient (odds ratio); 95% CI for EXP(B), 95% confidence interval for the odds ratio; EAR, estimated average requirement; BMI (m^2^/kg), body mass index (square meters per kilogram); AI, adequate intake
